# MR Electrical Properties Tomography Acquisitions: A Guideline From the ISMRM Electro‐Magnetic Tissue Properties Study Group

**DOI:** 10.1002/jmri.70057

**Published:** 2025-08-20

**Authors:** Stefano Mandija, Cornelis A. T. van den Berg, Ilias Giannakopoulos, Zhongzheng He, Yusuf Ziya Ider, Kyu‐Jin Jung, Nitish Katoch, Dong‐Hyun Kim, Riccardo Lattanzi, Paul Soullié, Ulrich Katscher

**Affiliations:** ^1^ Computational Imaging Group for MR Therapy and Diagnostic, University Medical Center Utrecht Utrecht the Netherlands; ^2^ Bernard and Irene Schwartz Center for Biomedical Imaging and Center for Advanced Imaging Innovation and Research (CAI^2^R), Department of Radiology, New York University Grossman School of Medicine New York New York USA; ^3^ IADI U1254, INSERM and Université de Lorraine Nancy France; ^4^ ICube (UMR 7357) and University of Strasbourg Strasbourg France; ^5^ Department of Biomedical Engineering Baskent University Ankara Turkey; ^6^ Department of Electrical and Electronic Engineering Yonsei University Seoul Republic of Korea; ^7^ Health Systems, Philips Healthcare Seoul Republic of Korea; ^8^ Philips Innovative Technologies Hamburg Germany

## 1 Introduction

The electrical properties of human tissue (i.e., electrical conductivity *σ* and permittivity *ε*) can be measured quantitatively in vivo with standard MR sequences and standard MR systems using a technique called Electrical Properties Tomography (EPT) [1, 2]. The full implementation of EPT involves first measuring the complex RF transmit field component B_1_^+^ (i.e., its magnitude |B_1_^+^| and phase ϕ^+^) as modulated by the tissue electrical properties, and then reconstructing *σ* and *ε* from the modulated B_1_^+^. The reconstructed electrical properties correspond to the Larmor frequency of the applied main field strength B_0_.

This article is meant as a short guideline for measuring complex B_1_^+^ for EPT. A comprehensive review on how to perform the subsequent reconstruction of *σ* and *ε* from the measured complex B_1_^+^ was reported earlier [3]. Software packages performing such reconstructions are available publicly [4, 5, 6], and so are EPT example datasets [7, 8].

This guideline will be complemented by two further EPT‐related guidelines, that is on standardization and phantom building, provided in separate articles. Together, these three guidelines are thought to parallel corresponding guidelines on mapping magnetic properties of tissue [9].

## 2 Measuring Complex B_1_

Although sequences with simultaneous measurements of |B_1_^+^| and ϕ^+^ are possible [10], current practice is to use separate measurements for |B_1_^+^| and ϕ^+^, as described in the following.

## 3 Measuring B_1_ ‐Magnitude

Numerous B_1_‐mapping techniques for measuring |B_1_^+^| have been published, independent of EPT (e.g., Actual Flip angle Imaging (AFI) [11], Bloch‐Siegert mapping [12], and Dual Refocusing Echo Acquisition Mode (DREAM) [13]). A comparison of B_1_‐mapping techniques on the determination of *ε* primarily highlighted the need for high SNR [14]. On the other hand, reviews of B_1_‐mapping techniques without reference to EPT were published [15].

Although relative units of |B_1_^+^| are sufficient for the majority of EPT approaches, such as differential equation‐based methods, absolute scaling of |B_1_^+^| (e.g., in μT) is required for other EPT approaches, like some integral equation‐based methods, as these rely on accurate field magnitudes to compute the necessary integrals.

## 4 Measuring B_1_ ‐Phase

A standard MR system does not allow ϕ^+^ to be measured. Consequently, most EPT studies make use of the so‐called transceive phase ϕ^±^, which is the superposition of the B_1_‐phases from RF transmission and RF reception, assuming ϕ^+^~ϕ^±^/2 (so‐called “Transceive Phase Assumption”, TPA). Thus, this subsection describes the measurement of ϕ^±^.

In principle, the phase image of any clinical sequence can be taken as ϕ^±^; however, it must be free of components unrelated to RF penetration. Unwanted phase contributions originate mainly from three sources: (A) B_0_‐inhomogeneities, (B) gradient‐induced eddy currents, (C) patient/tissue motion.

(A) Phase due to B_0_‐inhomogeneities can be avoided by using spin‐echo‐based sequences, gradient‐echo‐based sequences with balanced gradients, or sequences with ultrashort/zero TE [16]. Gradient‐echo‐based sequences without gradient balancing can be used to estimate ϕ^±^ if multiple echoes with different TEs are acquired and the observed phase for each voxel is extrapolated back to TE = 0 [17]. In the presence of chemical shift effects (e.g., due to fat), one might not be able to resolve the additional phase component.

(B) Unwanted phase due to eddy currents typically appears as artificially increased conductivity along one side of the field‐of‐view (FOV) and artificially decreased conductivity along the other side of the FOV. This too can be eliminated by using gradient‐echo‐based sequences with balanced gradients or by repeating spin‐echo sequences with inverted gradient polarization and averaging the results [18].

(C) As motion affects all types of sequences, standard motion correction schemes should be applied to minimize motion‐induced phase contributions. Due to the applied numerical derivatives, reconstructed maps are typically more corrupted by motion than the original scan's magnitude/phase image itself.

## 5 General Remarks

The optimal *field strength* B_0_ for conductivity mapping involves a trade‐off between sufficient SNR (i.e., high B_0_) and the validity of the TPA, which is increasingly violated with increasing B_0_ and object size. For brain applications (and with today's reported EPT techniques), B_0_ = 3T appears to provide a good trade‐off [18]. B_0_ = 1.5T might be a better choice for other in vivo applications, and B_0_ > 3T for imaging small animals. For permittivity, TPA plays a minor role, and the quest for optimal B_0_ is dominated by SNR, with accuracy increasing at least up to B_0_ = 7T [18]. Future studies for the upcoming systems with B_0_ > 7T might reveal an upper bound for optimal permittivity imaging.

The optimal *voxel size* for EPT is chosen by trading‐off sufficient SNR (i.e., large voxels) against the need for a sufficient number of voxels per spatial direction (i.e., small voxels) for stable numerical calculations of the Laplacian operator [19], which requires a minimum of *N* = 3 voxels per spatial direction within a tissue compartment. Although *N* = 3 is sufficient for an ideal noise‐free case, in realistic measurements including noise, *N* > 10 is frequently recommended. This aspect should be taken into account while defining the applied sequence's voxel size, as anatomic compartments smaller than *N* voxels cannot be reconstructed reliably. For conductivity studies, this consideration applies mainly to measuring ϕ^±^, as |B_1_^+^| acts here mainly as a spatially smooth correction term.

A *volumetric image* (i.e., a 3D or multi‐slice 2D scan) is required for the full version of EPT. 3D scans may be preferred as they avoid through‐plane inconsistencies when measuring ϕ^±^. Alternatively, the EPT reconstructions can be restricted to 2D in‐plane, thus eliminating through‐plane inconsistencies and introducing the additional model assumption of constant B_1_^+^ in the through‐plane direction.

If an *RF receive coil array* is used, care should be taken when combining the different array elements due to the possibility of violating the TPA [20]. RF transmission is based on quadrature (body or head) coils in the majority of EPT studies published yet.

*Acceleration techniques* are compatible with EPT, at least using moderate acceleration factors [21].

## 6 Summary—Recommendations

To support brain conductivity imaging, |B_1_^+^| can be acquired using low‐resolution B_1_‐mapping (e.g., voxel‐size = 4 (RO) × 8 (PE) × 8 (PE) mm^3^), followed by interpolation to the higher resolution of the ϕ^±^ map (see below). For permittivity imaging, a higher resolution of B_1_‐mapping is needed (e.g., voxel‐size = 2 × 2 × 2 mm^3^).

If available, low‐resolution B_1_‐mapping might be acquired using dual TR sequences (e.g., AFI with TR1/TR2 = 50/200 ms, TE = 2.3 ms, scan‐time = 2.3 min) [11], and high‐resolution B_1_‐mapping using dual TE sequences (e.g., DREAM with TR = 7 ms, TE1/TE2 = 2.5/3.4 ms, 2 averages, scan‐time = 2.2 min) [13]. Other vendor‐specific sequences may also be available. However, the specific choice of the B_1_‐mapping sequence does not seem to be of utmost importance, as long as the main demand for high SNR is fulfilled [14].

To image ϕ^±^, we recommend a 3D acquisition (e.g., 3D balanced Fast Field Echo (bFFE)/balanced Steady State Free Precession (bSSFP), voxel‐size = 1 × 1 × 1 mm^3^, TR = 3 ms, TE = 1.5 ms, dynamics = 2, scan‐time = 3.5 min). We recommend the acquisition of multiple, separate dynamics rather than signal averages to better resolve artifacts arising for example from flow, and allow co‐registration to reduce motion effects. Potential banding artifacts might be reduced via phase cycling or acquisitions with different TR. A 2D acquisition may be used if fast temporal changes in conductivity are to be examined.
